# Reactive Oxygen Species in Chronic Obstructive Pulmonary Disease

**DOI:** 10.1155/2018/5730395

**Published:** 2018-02-11

**Authors:** Samia Boukhenouna, Mark A. Wilson, Karim Bahmed, Beata Kosmider

**Affiliations:** ^1^Department of Thoracic Medicine and Surgery, Temple University, Philadelphia, PA 19140, USA; ^2^Center for Inflammation, Translational and Clinical Lung Research, Temple University, Philadelphia, PA 19140, USA; ^3^Redox Biology Center and Department of Biochemistry, University of Nebraska, Lincoln, NE 68588, USA; ^4^Department of Physiology, Temple University, Philadelphia, PA 19140, USA

## Abstract

Chronic obstructive pulmonary disease (COPD) includes chronic bronchitis and emphysema. Environmental exposure, primarily cigarette smoking, can cause high oxidative stress and is the main factor of COPD development. Cigarette smoke also contributes to the imbalance of oxidant/antioxidant due to exogenous reactive oxygen species (ROS). Moreover, endogenously released ROS during the inflammatory process and mitochondrial dysfunction may contribute to this disease progression. ROS and reactive nitrogen species (RNS) can oxidize different biomolecules such as DNA, proteins, and lipids leading to epithelial cell injury and death. Various detoxifying enzymes and antioxidant defense systems can be involved in ROS removal. In this review, we summarize the main findings regarding the biological role of ROS, which may contribute to COPD development, and cytoprotective mechanisms against this disease progression.

## 1. Introduction

**Figure 1:**
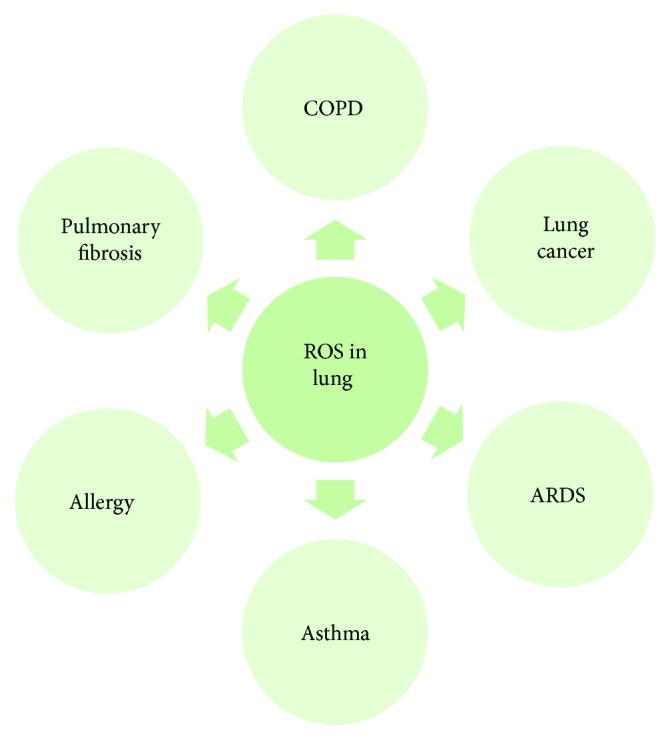
Potential contribution of ROS to various lung disease development. ROS—reactive oxygen species; COPD—chronic obstructive pulmonary disease; ARDS—acute respiratory distress syndrome.

Chronic obstructive pulmonary disease (COPD) is a major health problem that is becoming the leading cause of morbidity and mortality throughout the world [1]. This disease is characterized by chronic inflammation, remodeling of the small airways, and destruction of the lung parenchyma [2]. It is believed that oxidative stress is increased in patients with COPD due to chronic exposure to cigarette smoke, a main risk factor, which contains a high concentration of oxidants and reactive oxygen species (ROS) (Figure 1) [3]. Other factors can also contribute to COPD development, such as bacterial and viral infections. Disease development is linked to a protease/antiprotease imbalance [4] that may lead to the lack of the protection against elastolytic enzymes. This imbalance may also create the disproportion of oxidant/antioxidant due to high endogenous ROS released by inflammatory cells such as neutrophils, macrophages, and structural cells, for example, epithelial and endothelial cells [1]. However, cells can be protected against oxidative stress by enzymatic and nonenzymatic antioxidant systems [5]. Preclinical studies and clinical trials have shown that antioxidant molecules such as small thiol molecules (N-acetyl-L-cysteine and carbocysteine) [6–8], antioxidant enzymes (glutathione peroxidases) [9], activators of Nrf2-regulted antioxidant defense system (sulforaphane) [10, 11], and vitamins, for example, C, E, and D [12–14], can boost the endogenous antioxidant system and reduce oxidative stress. In addition, they may slow the progression of COPD. In this review, we focus on the mechanism of action of endogenous and exogenous ROS that can contribute to this disease development and the cytoprotective role of antioxidant molecules [15].

## 2. Chronic Obstructive Pulmonary Disease

COPD is the fourth leading cause of death in the United States [16] and is set to become the third cause of mortality in 2020 worldwide [17]. COPD is as a common, preventable, and treatable disease, characterized by persistent airflow limitation that is usually progressive and associated with an enhanced chronic inflammatory response in the airways and the lung to noxious particles or gases. The most commonly encountered risk factor for COPD is cigarette smoke [2]. Moreover, outdoor, occupational, and indoor air pollution may contribute to this disease development. COPD refers mainly to two types: chronic bronchitis and emphysema. Chronic bronchitis is defined as the presence of a cough and sputum production for at least three months in each of two consecutive years [16, 18]. Emphysema is characterized by the destruction of the alveoli, the tiny air sacs in the lung where the exchange of oxygen and carbon dioxide takes place, which results in a decreased level of oxygen in the blood (hypoxemia) combined with an increased level of carbon dioxide in the blood (hypercapnia). Tuder et al. [19, 20] indicated that cigarette smoke could induce alveolar wall destruction by the interaction of apoptosis, oxidative stress, and protease/antiprotease imbalance. This may cause emphysema, which leads to the progressive and relentless loss of lung function due to the destruction of lung parenchyma and chronic inflammation. Furthermore, studies from animal models indicate that 4- to 6-month exposure to cigarette smoke leads to emphysema development in mice, rats, and rabbits [21–23]. Exacerbations of COPD are of major global importance [24]. Exacerbations are defined as sustained worsening of the patient's condition of the stable state and beyond normal day-to-day variations that is acute in onset and may warrant additional treatment in a patient with underlying COPD [25]. It has been reported that exacerbations are also involved in emphysema progression in patients with COPD [26]. Bacteria, viruses, and environmental agents account for the vast majority of episodes of exacerbation. Exacerbation, systemic inflammation, ROS generation, alterations of metabolism, cardiovascular events, and lung cancer contribute to the overall disease severity and untimely death [2, 20].

## 3. Oxidative Damage of Biological Molecules

**Figure 2:**
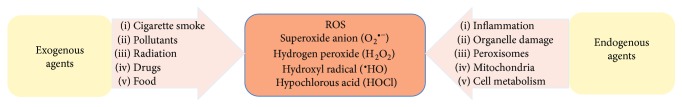
Exogenous and endogenous sources of ROS such as superoxide anions, hydrogen peroxide, hydroxyl radicals, and hypochlorous acid in cells.

**Figure 3:**
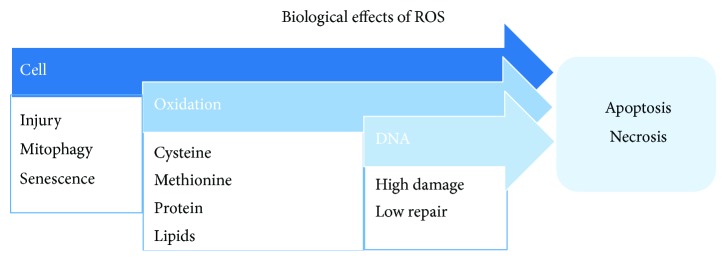
ROS reaction with various biomolecules such as proteins, lipids, and DNA may cause cell injury leading to apoptosis and necrosis.

Exposure to exogenous sources of ROS such as cigarette smoke, air pollutants, or endogenously released ROS from leukocytes and macrophages involved in the inflammatory process can induce oxidative stress and the oxidant/antioxidant imbalance (Figure 2) [15]. Neutrophils have a key role in inflammatory processes and have been implicated in the development and progression of all of the pulmonary features of COPD through the release of destructive mediators such as neutrophil elastase and matrix metalloproteinases. Moreover, pulmonary neutrophilic inflammation is a feature of cigarette smoking, but importantly, in patients with COPD, it is sustained even following smoking cessation [27, 28]. Activated immune cells such as neutrophils and macrophages release ROS as a part of the inflammatory process [29]. ROS can react with biological molecules such as lipid, protein, DNA, RNA, and mitochondrial DNA and leads to epithelial cell injury and death (Figure 3), which contribute to COPD development.

**Figure 4:**
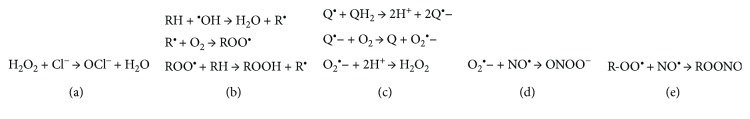
The mechanism of ROS interaction with biomolecules. (a) Hypochlorite anion production catalyzed by myeloperoxidase; (b) lipid peroxidation; (c) production of hydrogen peroxide; (d) peroxynitrite generation; (e) production of alkyl peroxynitrites. H_2_O_2_—hydrogen peroxide; ^−^OCl—hypochlorite anion; RH—unsaturated lipid; ^•^OH—hydroxyl radical; R^•^—lipid radical; ROO^•^—lipid peroxyl radical; ROOH—lipid peroxide; Q/QH_2_—quinone/hydroquinone; O_2_^•−^—superoxide anion; NO^•^—nitric oxide; ONOO^−^—peroxynitrite; ROONO—alkyl peroxynitrites.

During the respiratory burst, neutrophil myeloperoxidase catalyzes the oxidation of chloride ions (Cl^−^) by hydrogen peroxide (H_2_O_2_) to generate the anionic ROS hypochlorite (OCl-) or its conjugate acid, hypochlorous acid (HOCl) (Figure 4(a)) [27]. The concentration of HOCl in the interstitial fluids of inflamed tissue has been estimated to reach more than 5 mM. HOCl has high reactivity, rapidly reacts with a variety of biomolecules, and cannot reach distant intracellular targets [30]. However, reaction of HOCl with amines can generate much more stable chloramines that can diffuse greater distances [27]. Only a few low molecular weight amines, such as nicotine in cigarette smoke, have been found to form chloramines that can cross cellular membranes and mediate HOCl-induced intracellular protein damage [31].

At the molecular level, ROS may induce lipid peroxidation (Figure 4(b)) and yield products such as malondialdehyde, which has the ability to inactivate many cellular proteins by generating protein cross-linkages [32]. This may stimulate pulmonary inflammation [33], promoting alveolar wall destruction and emphysema development. Another product of lipid peroxidation is 4-hydroxy-2,3-nonenal, which has many cytotoxic effects [34]. It has been shown to cause cytoplasmic Ca^2+^ accumulation, induce expression of proinflammatory cytokines and NF-*κ*B, mitochondrial dysfunction, and apoptosis. The end products of lipid peroxidation such as ethane, pentane, and 8-isoprostane are elevated in the breath and serum of patients with COPD [35].

ROS can also cause reversible and irreversible protein modifications. Protein s-sulfenation, s-nitrosylation, s-glutathionylation, disulfides, thiosulfinates, sulfenamides, sulfinamides, and persulfides are reversible modifications [36, 37]. They are involved in redox regulation of protein functions by ROS and RNS. Moreover, these modifications play important roles in health because they contribute to regulation of cellular defense systems and protection against oxidative stress. Protein carbonyls, nitrotyrosines, sulfinic acids, sulfonic acids, and sulfonamides are irreversible modifications [37, 38]. Oxidation of proteins may lead to activation of NF-*κ*B, p38 MAPK, induction of inflammatory genes, and inhibition of the activity of endogenous antiproteases, which may contribute to this disease pathogenesis [39]. Although, irreversibly oxidized proteins are often indicators of high oxidative stress and oxidative damage and are detected in lung diseases, they may also be present under normal conditions.

Moreover, ROS can also induce RNA, DNA, and mitochondrial DNA (mtDNA) damage. Studies suggest that RNA is more vulnerable to oxidative damage than other cellular components [40]. RNA could have enhanced susceptibility for oxidative attack because of its widespread cytosolic distribution, single-stranded structure, absence of protective histones, and lack of an advanced repair mechanism [41]. More than 20 different types of base damage by hydroxyl radicals have been identified [40].

The most prevalent oxidized base in RNA is 8-hydroxyguanosine (8-OHG). The highly reactive hydroxyl radical first reacts with guanine to form a C8-OH adduct radical. Then, the loss of an electron and proton generates 8-OHG (an oxidized RNA nucleoside). It is worth to notice that RNA oxidation is more prevalent than DNA oxidation in alveolar wall cells in emphysema [41]. However, DNA oxidation promotes microsatellite instability, inhibits methylation, and accelerates telomere shortening. 8-hydroxy-2′-deoxyguanosine (8-OHdG) is a product of oxidized DNA and widely used as a marker of oxidative cellular damage. Moreover, p53 mutation, observed in lung cancer, is linked to a direct DNA damage due to exposure to carcinogens in cigarette smoke [42]. It is worth noting that patients with emphysema have a high risk of lung cancer development [43]. ROS are also the main source of mtDNA damage and mutagenesis [44]. The main products of mtDNA base damage are thymine glycol among pyrimidines and 8-OHdG among purines. The former has low mutagenicity, whereas the latter upon replication can cause characteristic G→T transversions. MtDNA with oxidative damage may lead to mitochondrial dysfunction in alveolar epithelial cells [45].

## 4. Reactive Oxygen Species

It is believed that oxidative stress induced by cigarette smoke and oxidative cell damage play a pivotal role in the COPD development [7]. Cigarette smoke is a complex mixture of numerous free radicals and ROS that can be divided into two phases: tar (particle) and gas. Tar phase covers about 10^17^ relatively long-lived radical molecules per gram, for example, quinone/hydroquinone (Q/QH_2_) radicals that can reduce oxygen to produce superoxide anion (O_2_^•−^) leading the generation of H_2_O_2_ (Figure 4(c)) and hydroxyl radical (^•^OH) [3, 46]. Primary among highly reactive ROS is ^•^OH, which can damage all types of macromolecules upon collision, thus having a diffusion-limited lifetime of approximately 1 nanosecond [47]. Hydroxyl radicals can be generated by Fenton chemistry involving H_2_O_2_ and either ferrous iron (Fe(II)) or cuprous copper (Cu(I)), which constitute dangerous intersections of metal and redox homeostasis. Particulate matter (PM) pollutants were shown to be iron-rich and to increase oxidative stress, providing opportunity for damaging Fenton chemistry to occur and generate ^•^OH in the lung [48]. Less reactive than ^•^OH but still dangerous is the superoxide radical anion (O_2_^•−^), which can participate in one electron redox chemistry, predominantly with metals and flavin cofactors. In contrast, H_2_O_2_ is a relatively stable, neutral ROS that can diffuse significant distances from its site of production [47]. Unlike the superoxide radical anion, H_2_O_2_ participates primarily in two-electron redox chemistry, predominantly with sulfur-containing moieties in the cell. However, H_2_O_2_ can also participate in some one-electron chemistry with transition metals (see above for a discussion of Fenton reaction). H_2_O_2_ can serve as a signaling molecule at low concentrations as well as a damage agent at higher concentrations and thus has a complex cellular role that is defined by overlapping mechanisms of H_2_O_2_ detection, signal transduction, and destruction [49, 50]. Moreover, HOCl generated in the presence of H_2_O_2_ can further lead to formation of more toxic ROS such as ^•^OH [27]. The high reactivity of the hypochlorite anion (^−^OCl) means that it is fairly indiscriminate in modifying its targets, typically with second order rate constants of 10^5^–10^7^ M^−1^ s^−1^ [51]. In proteins, cysteine, histidine, and methionine are among the favored residues for modification. Primary amines, such as those found in the sidechain of lysine, can also be modified to chloramines by ^−^OCl. In total, high ROS levels may cause lung tissue damage and respiratory problems via modification of diverse target molecules via distinct, ROS-specific mechanisms.

Gas phase of cigarette smoke contains much more reactive molecules than tar. This phase consists of 10^15^ organic and inorganic radicals per puff [3], for example, nitric oxide (NO^•^), nitrogen dioxide, and peroxynitrite (ONOO^−^). Cigarette smoke contains 74.5–1008 ppm NO^•^ [52] and thus represents one of the main ROS and reactive nitrogen species (RNS) to which smokers are exposed. NO^•^ has a short half-life (t_1/2_~0.09 to 2 s) [53]; however, it reacts quickly (second order rate constant ~2.4 ± 0.3 × 10^6^ M^−2^ s^−1^) [54] with O_2_^•−^ to form peroxynitrite (ONOO^−^) (Figure 4(d)). Peroxynitrite is a RNS that is involved in many physiological and pathological processes [55, 56]. Peroxynitrite possesses a very strong oxidation and nitration capabilities, leading to damaging molecules in cells, such as DNA and proteins. A second order reaction depends on the concentrations of one second order reactant or two first order reactants, which are O_2_^•−^ and NO^•^ in the case of peroxynitrite generation. NO^•^ can also react with organic lipid peroxyl radicals (ROO^•^) present in cigarette smoke to form alkyl peroxynitrites (ROONO) (Figure 4(e)), which are cytotoxic species. Moreover, NO^•^ and O_2_^•−^ are produced by inflammatory cells such as macrophages, by nitric oxide synthases (NOSs) and NADPH oxidase complexes (NOXs), respectively. Furthermore, ROS and RNS can be released by a noncontrolled process as by-products during mitochondrial respiration, peroxisomal metabolism [57], and protein folding maturation process in the endoplasmic reticulum [58]. Their increased formation leads to oxidative stress and lung damage.

## 5. Mitochondrial Dysfunction

Mitochondria are dynamic intracellular organelles that constantly change in shape, size, number, and distribution through constitutive cycles of fusion and fission [59]. Mitochondrial fusion contributes to maintain intact mitochondrial DNA copies, mitochondrial membrane components, and matrix metabolites. Mitochondrial fission plays a role in the segregation of dysfunctional mitochondria from the pool of mitochondria. Accordingly, mitochondrial fission is highly correlated with cell apoptosis [60]. Specifically, mitochondrial fission is achieved by phosphorylation of Drp1 at Ser616, which promotes the recruitment of Drp1 from the cytosol to the mitochondrial surface by human fission protein-1. The possible mechanism indicates that oxidative stress triggers mitochondrial fission and loss by enhancing Drp1 translocation from the cytosol. Cigarette smoking-induced mitochondrial ROS can accelerate phosphorylation of Drp1. Therefore, prolonged oxidative stress can cause an imbalance in fission-fusion, resulting in mitochondrial fragmentation, which may contribute to cell death.

Mitochondria may serve as sensors to detect perturbations of intracellular homeostasis, including oxidative stress [61]. Histone proteins are reported to protect DNA from a variety of potentially dangerous ROS, such as ^•^OH. High sensitivity of mtDNA to damage caused by oxidative stress is related to the proximity to the source of ROS, the lack of protective histones, and a relatively inefficient mtDNA repair [62]. This may induce the synthesis of defective mitochondrial electron transport chain subunits, further resulting in the decreasing transmembrane potential and leading to the abnormal overproduction of ROS, which damage cells [59]. This further contributes to disturbances in the redox balance leading to the imbalance between the oxidants and antioxidants in the cell. Finally, this cause mitochondrial dysfunction, permeabilization of the outer mitochondrial membrane, release of apoptotic proteins, and cell death [63]. Specifically, O_2_^•−^ can lead to mitochondrial depolarization by facilitating cytochrome c release. Mitochondrial dysfunction has been reported in airway smooth muscle cells obtained from smokers and patients with COPD [64]. These cells were unable to provide adequate respiration and had a severely reduced respiratory reserve capacity. Bronchial epithelial cells obtained from ex-smokers with COPD showed damaged mitochondria, with depletion of cristae, increased branching, elongation, and swelling [65]. Moreover, mitochondrial dysfunction in patients with COPD is associated with excessive mitochondrial ROS levels, which contribute to enhanced inflammation [64].

Damaged or dysfunctional mitochondria are cleared from the cells by the autophagy-dependent turnover of mitochondria (mitophagy) [66]. Mitophagy is considered a homeostatic program that maintains a healthy mitochondrial population for cytoprotective roles in disease pathogenesis [67]. In contrast, mitophagy may be also a possible effector of cell death programs. Recent studies indicate that mitophagy is associated with epithelial cell death in COPD, specifically involving necroptosis, a form of programmed necrosis, in response to cigarette smoke exposure. In cultured pulmonary epithelial cells, cigarette smoke caused mitochondrial dysfunction associated with a decline of mitochondrial membrane potential and increased mitochondrial ROS production. Furthermore, it was reported that mild and transient oxidative stress induced by H_2_O_2_ does not damage mitochondria, but rather initiates a ROS signaling cascade, leading to the induction of selective mitophagy [68]. This in turn would promote the selective removal of damaged mitochondria. Prolonged and more excessive ROS triggers early phase of autophagic process, including cytoprotection. However, higher ROS concentrations may overload this and other quality control systems, leading to permanent cell damage and reduced viability. Based on these observations, ROS can act as signaling molecules influencing cell fate. Redox regulation can promote both survival, for example, during starvation. On the other hand, if the prosurvival attempt fails, high oxidative stress causes cell death [69]. Taken together, studies suggest that mitochondrial dysfunction induced by oxidative stress is a key contributor to the pathophysiology of COPD. Targeting mitochondrial ROS represents a promising therapeutic approach in patients with this disease.

## 6. Antioxidant Defenses against ROS

Cells mount a diverse and robust defense against ROS, which includes an overlapping array of enzyme activities that are specific for particular ROS. Of the common ROS, only ^•^OH is so reactive that there are no effective enzymatic detoxification strategies (see above for a discussion of ^•^OH). Because ^•^OH is so indiscriminately destructive, no mechanisms in the cell can effectively counter it. Although glutathione (GSH) has been suggested as a general redox buffer against this and other ROS, recent work suggests that GSH exerts its antioxidant effect mostly through enzymatic pathways such as glutaredoxins or as the reductant for glutathione peroxidases [70]. Tocopherol and ascorbate form moderately stable radicals and thus can act as “sinks” for ^•^OH and other radicals, although ^•^OH will react with the first molecule it encounters, which is unlikely to be a small molecule antioxidant.

**Figure 5:**
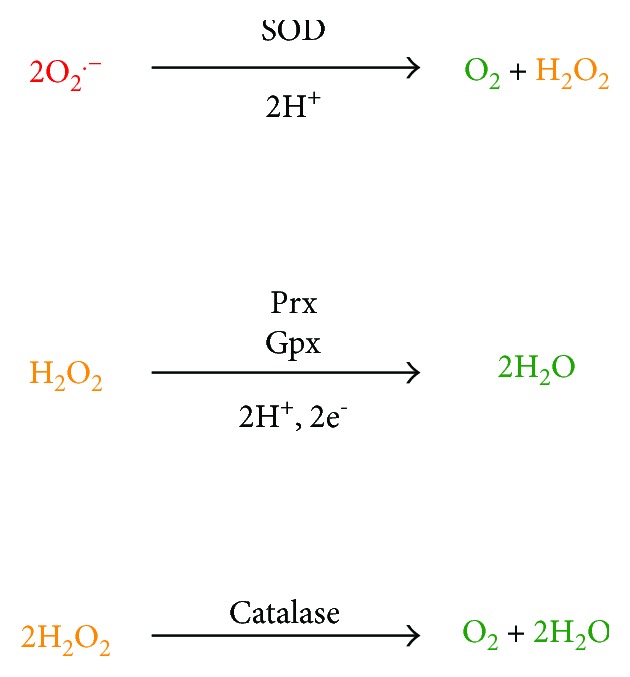
Primary enzymatic means of ROS detoxification. The relative reactivity of the ROS is indicated by color, ranging from highly reactive (red) to inert (green). SOD—superoxide dismutases; Prxs—peroxiredoxins; Gpxs—glutathione peroxidases.

Another radical ROS is O_2_^•−^. The superoxide anion is detoxified by the action of the metalloenzyme superoxide dismutase (SOD), which converts O_2_^•−^ to H_2_O_2_ and O_2_ (Figure 5).

Being a dismutation, there is no net change in redox state and thus no electrons are required for the balanced reaction [71]. Various SODs exist and are classified according to the metals present in their active sites, which in humans are the Cu-Zn SODs (SOD1; intracellular, SOD3; extracellular) and Mn-SOD (SOD2, mitochondrial). Notably, the Cu-Zn and Mn-SODs have completely different three-dimensional structures as a consequence of being members of different fold families, suggestive of convergent evolution [72]. SODs are highly efficient enzymes, dismutating O_2_^•−^ with a k_cat_/K_M_~7 × 10^9^ M^−1^ s^−1^, which exceeds the “diffusion limit” of ~10^9^ M^−1^ s^−1^. Enzymes operating at the diffusion limit successfully catalyze their reaction nearly every time they encounter substrate (i.e., they are catalytically “perfect”), which would seem to place an upper limit on enzyme catalytic efficiency. However, because O_2_^•−^ is an anion, the electrostatic properties of SOD are important and have been evolutionarily optimized to guide this negatively charged substrate toward a positively charged patch near the SOD active site. In this manner, SOD electrostatically “funnels” substrate toward its active site and thus exceeds the theoretical diffusion limit on catalytic efficiency [73]. Therefore, SOD is remarkably proficient at removing O_2_^•−^, although one of its products is H_2_O_2_, another ROS. Extracellular O_2_^•−^ produced by neutrophils is considered a major source of alveolar and bronchial epithelial cell damage, and the SOD system is an important component in the pulmonary defense against hyperoxic injury [74, 75].

The predominant enzymes that handle hydrogen peroxide are the peroxiredoxins (Prxs), a collection of thiol-dependent enzymes that convert peroxides into water (when H_2_O_2_ is the substrate; Figure 5) or alcohols (when organic peroxides of the general formula ROOH are the substrate). Their high cellular concentration and fast rate of reaction of Prxs with peroxides (k_cat_/K_M_~10^6^-10^7^ M^−1^ s^−1^) mean that they are likely the first molecules in the cell that react with this ROS [76]. Therefore, Prxs have been postulated to be both the front-line defense against elevated peroxide and to mediate initial homeostatic or proliferative peroxide signaling events [77]. In addition to their role in directly detoxifying H_2_O_2_, the Prxs also indirectly decrease the levels of hypochlorite (^−^OCl) and hydroxyl radical (^•^OH) by reducing the concentration of the peroxide reactant that generates these secondary ROS.

There are six Prxs in mammals, divided into 1-Cys or 2-Cys classes depending on the number of critical cysteine residues in their active sites. Regardless of class, all Prx reduce peroxides via the initial formation of a cysteine sulfenic acid (Cys-SOH) at a highly reactive, peroxidatic cysteine active site residue. The peroxide-derived oxygen atom of the cysteine sulfenic acid intermediate is released as water during the resolution of the sulfenic acid by the attack of a second thiol, either donated by another cysteine residue in the protein (the resolving cysteine in the 2-Cys Prxs) or from small molecule thiols such as glutathione (in 1-Cys Prxs). Therefore, Prx catalysis results in disulfide-containing enzymes that must be reduced by thioredoxin or glutaredoxin in order to restore the resting enzyme and complete the catalytic cycle. This process is dependent on reductases that ultimately obtain electrons from NADPH, thereby coupling Prx-dependent ROS detoxification to the pentose phosphate pathway, which generates NADPH. Prxs I, II, III, and V are the isoforms that are most highly expressed in healthy lung epithelium [78]. Additional evidence suggests that the 1-Cys Prx VI is important specifically for the defense against lipid peroxides in the lung [79]. A further point of particular interest is that Prx VI also possesses phospholipase A2 activity and it is thought to play an important role in the metabolism of lung surfactant phospholipids that appears to be independent of its peroxidase function.

Glutathione peroxidases (Gpxs) are an intriguing class of oxidative stress defense enzymes that typically (though not always) feature a selenocysteine residue in their active sites. Selenocysteine, sometimes called the 21st amino acid, is sometimes found in the active sites of redox-active enzymes [80]. The Gpxs detoxify peroxides using a catalytic strategy that is broadly similar to the Prxs (see above), involving the transient oxidation of an active site residue (cysteine in the Prxs, selenocysteine in the Gpxs) to a monooxygenated form, either cysteine-sulfenic acid (Cys-SOH) in the Prxs or selenocysteine selenenic acid (Sec-SeOH) in the Gpxs. In the Gpxs, this Sec-SeOH is resolved by the sequential action of two molecules of GSH. The first GSH attacks the Sec-SeOH to form a Se-S bond with the enzyme and liberates water (Figure 5). The second GSH regenerates the free enzyme and produces oxidized glutathione disulfide (GSSG) with the first GSH. As such, Gpx activity is critically linked to the cellular glutathione pool, and the released GSSG is reduced to GSH by NADPH-dependent glutathione reductase. Interestingly, Gpx1 deficiency results in only lung modest phenotypes in mouse models; however, alteration of pulmonary immune function has been noted [81]. In general, it is likely that the multiple Prx and Gpx isoforms present in mammals have significantly overlapping activities and that redundancy has evolved in the biochemical mechanisms for removing peroxides.

A curiosity of the cellular defense against peroxides is that it contains so many enzymes apparently dedicated to this task. Catalases are also hydrogen peroxide detoxifying enzymes that, unlike the Prxs and Gpxs, use a reductase-independent, hemedependent chemistry to convert H_2_O_2_ to O_2_ (Figure 5). The hemedependent peroxidases are a related family of enzymes that convert H_2_O_2_ to water, and some enzymes have both activities in a single polypeptide. Catalases are fast enzymes, with k_cat_ values of ~10^7^ s^−1^ but also have very high K_M_ values of ~1 M for peroxide. Therefore, catalases are far from their maximum rate when presented with the nM-*μ*M levels of H_2_O_2_ present in cells and are likely to be kinetically outcompeted by the Prxs. This may result in differing kinetic regimes in which the Prxs and catalases operate, allowing effective response over a range of peroxide insult, from chronic low level (Prxs) to acute high level stress (catalases) [82]. Catalases are expressed in alveolar epithelial cells and may play a particularly important role in acute stress caused by bolus H_2_O_2_ generation, which occurs during reoxygenation injury in the lung [83].

## 7. Lung Aging

The ability to prevent the oxidative damage to the lung tissue and the potential to regenerate injured cells are two key determinants of aging [84]. While still an area of controversy, reports indicate that exposure to cigarette smoke, ROS, and other environmental stressors may accelerate biological processes associated with normal aging [85, 86]. Moreover, recent epidemiological study has suggested that about half of patients with COPD fail to achieve full lung function in adolescence and early adulthood. In these individuals, this disease might develop as a consequence of the “normal” decline in lung function with age. Similar to emphysema, lung aging is characterized by a decrease in the density and an increase in the diameter of the membranous bronchioles. However, unlike emphysema, there are no differences in alveolar attachments. COPD may represent an accelerated (or normal) form of lung aging.

## 8. Conclusions

We highlighted how environmental exposure to cigarette smoke and endogenous ROS generated during inflammatory processes induce high oxidative stress, which may contribute to COPD development. We summarized the reactivity of the most biologically relevant ROS and RNS, which can oxidize different biomolecules such as DNA, proteins, and lipids. We also reviewed how oxidant molecules (ROS) can be reduced or destroyed by diverse cytoprotective mechanisms focusing on the enzymatic protection afforded by SODs, Prxs, Gpxs, and catalases. Antioxidant systems, for example, GSH, vitamins A, C, and E, and carotenoids, are important and can intersect with other pathways [39]. Under very high oxidative stress conditions present in patients with COPD, these mechanisms may not correctly play their protective role, which may contribute to exacerbation.
